# Variability of trace elements in bodies of scrapers (Ephemeroptera) and predators (Plecoptera) from mountain rivers of Dzungarian Alatau (Kazakhstan) and Western Carpathians (Slovakia)

**DOI:** 10.1007/s11356-024-35527-5

**Published:** 2024-11-12

**Authors:** Jaroslav Solár, Martina Haas, Patrik Pánik, Berikzhan Oxikbayev, Aibek Abduakassov

**Affiliations:** 1 Institute of High Mountain Biology, University of Žilina, Tatranská Javorina 7, Tatranská Javorina, 059 56 Slovakia; 2 Zhetysu State University named after Ilyas Zhansugurov, Zhansugurov st. 187 A, 040009 Taldykorgan, Kazakhstan

**Keywords:** Freshwater benthic macroinvertebrates, Feeding groups, Bioaccumulation, Trace elements, Mountain streams

## Abstract

Bioaccumulation of trace elements in aquatic environments can be influenced by local environmental conditions such as temperature fluctuations, pH levels, sediment composition, dissolved organic matter content, and the presence of other chemical substances. We analyzed the differences in trace elements accumulation (S, Cl, K, Ca, Ti, Cr, Mn, Fe, Cu, Zn, Rb, Sr, Mo, Ba, and Pb) between two trophic guilds—scrapers (Ephemeroptera) and predators (Plecoptera)—of freshwater benthic macroinvertebrates collected from mountain streams in Kazakhstan and Slovakia. Trace elements in dried insect bodies were analyzed using an X-ray spectrometer, and physicochemical parameters of stream water were investigated at each sampling site. Our results showed significant differences in Fe, Ti, and Sr levels in predators from Kazakhstan and Cu levels in predators from Slovakia. Despite some trace elements showing higher concentrations in one group over another, the overall differences between regions were more pronounced. Principal component analysis (PCA) revealed that the primary factors influencing trace elements variability were associated with environmental conditions such as temperature, oxygen levels, and total dissolved solids (TDS). PCA components indicated a higher load of trace elements in the warmer, less oxygenated streams, particularly in Kazakhstan. These findings suggest that both biotic (feeding strategies) and abiotic (geographical and environmental conditions) factors significantly influence trace elements dynamics in freshwater ecosystems.

## Introduction

Trace elements enter aquatic environments, including mountain streams, from a variety of natural and anthropogenic sources. These sources are influenced by factors such as local climate, bedrock structure, topography, and land cover/use (Sajdak et al. 2018; Radecki-Pawlik et al. 2020). Generally, the total amount of basic trace elements in mountain streams (fed by precipitation) increases with decreasing elevation (e.g., Żelazny and Siwek 2012). Water temperature and flow rate are essential for the process of water mineralization (Todd et al. 2012; Manning et al. 2013; Shinohara et al. 2021). The availability of trace elements varies at each site due to the chemistry of the water (Di Toro et al. 2001; Mebane et al. 2020a), variability in hydroclimatic processes, heterogeneous conditions in mountainous environments, and pollution from nearby or long-range sources. The concentrations of the trace elements and the volume of suspended sediments display clear seasonal patterns in stream water (Bucher et al. 2017; Hamid et al. 2020). Therefore, considering the effective bioavailability of trace elements, aquatic biota may provide better insight into the long-term effects of contamination on aquatic communities and offer more accurate assessments of ecological water quality (Pastorino et al. 2019).

Freshwater benthic macroinvertebrates play crucial roles in aquatic ecosystems by participating in various trophic functions, including nutrient cycling, serving as food sources, and contributing to biological filtering and primary production (Collier et al. 2016). Higher concentrations of trace elements in their environment can reduce the abundance of sensitive species (e.g., Ephemeroptera and Plecoptera), suppress species diversity, or shift community composition towards more tolerant taxa (Kotalik and Clements 2019; Lidman et al. 2020; Mebane et al. 2020b). Consequently, freshwater benthic macroinvertebrates are widely used as indicators of biological conditions (Birk et al. 2012; Namba et al. 2020) and for water quality assessment (Feio et al. 2021; Vitecek et al. 2021). The main routes of trace elements uptake depend on an organism’s ecological interactions with its environment and its food resources (Luoma and Rainbow 2008). Trace element accumulation is typically higher in the bodies of freshwater benthic macroinvertebrates than in water and lower than in sediments or periphyton (Goodyear and McNeill 1999). This suggests that one key exposure route for accumulation of trace elements is the assimilation efficiency of elements from ingested food (Wang and Fisher 1999; Cain et al. 2011). Feeding habits appear to be the most important exposure route (Jones et al. 2020). Thus, the process of trace elements accumulation in freshwater benthic macroinvertebrates can be based on their functional feeding preferences (e.g., scrapers, predators as per Cummins and Klug 1979), and differences in trace elements accumulation can be expected among individual trophic guilds (Rodriguez et al. 2018; Pastorino et al. 2019, 2020a). Some elements (e.g., heavy metals) have an affinity for fine particulate organic matter and biofilms (Priya et al. 2022), leading to higher element loads in the bodies of macroinvertebrates that consume basal resources compared to predators (Santoro et al. 2009; Arnold et al. 2021). In scrapers, elements can be bioaccumulated mainly through the consumption of biofilm (e.g., algae, bacteria, or fungi) adhered to the surfaces of rocks and decaying vegetation in streams (Ponsatí et al. 2016; Hua et al. 2019; Fenoglio et al. 2020; Loureiro et al. 2021). The bioaccumulation of elements within the food web of freshwater benthic macroinvertebrates is driven by various environmental factors (Ubando et al. 2021). Generally, the mineralogical composition of sediments (Miranda et al. 2021) and temperature increase the bioavailability and mobility of elements (Li et al. 2023; Adams et al. 2020). Other important factors include stream flow and physicochemical conditions (e.g., pH, oxygen) which can induce higher or lower exchange rates of elements from sediment to water and biota (Brezonik et al. 1991; Wojtkowska et al. 2016; Kolarova and Napiórkowski 2021; Iordache et al. 2022). Despite the expected differences in specific stream and river conditions, including the bioavailability of elements, freshwater benthic macroinvertebrates may have a consistent response to trace elements accumulation. Further investigation is required as understanding accumulation patterns helps evaluate potential trace elements toxicity to freshwater benthic macroinvertebrates and, by extension, to other organisms at higher trophic levels in the ecosystem (Pastorino et al. 2019).

In this study, we analyzed the differences in trace elements accumulation between two trophic guilds of Ephemeroptera (scrapers) and Plecoptera (predators) species with respect to their functional feeding preferences. We focused on mountain streams and rivers of the Dzungarian Alatau (Kazakhstan) and the Western Carpathians (Slovakia), which are not directly affected by human activities, to avoid the effects of pollution. Additionally, we analyzed trace elements accumulation in their bodies with respect to the physicochemical properties of the water. The objectives of the study were (1) to test whether trace elements accumulation differs between trophic levels (scrapers vs. predators) of freshwater benthic macroinvertebrates, (2) to determine whether these differences are similar between the study areas, and (3) to assess whether these differences correlate with basic physicochemical properties of the streams. Comparing trace elements accumulation patterns between different study areas (Dzungarian Alatau and Western Carpathians), despite differences in sediment mineralogical composition, provides valuable insights into the universality or context-specificity of these patterns. Similar studies have not made such cross-regional comparisons. Such comparisons can contribute to a more comprehensive understanding of the factors driving trace elements dynamics in mountain stream ecosystems.

## Materials and methods

**Fig. 1:**
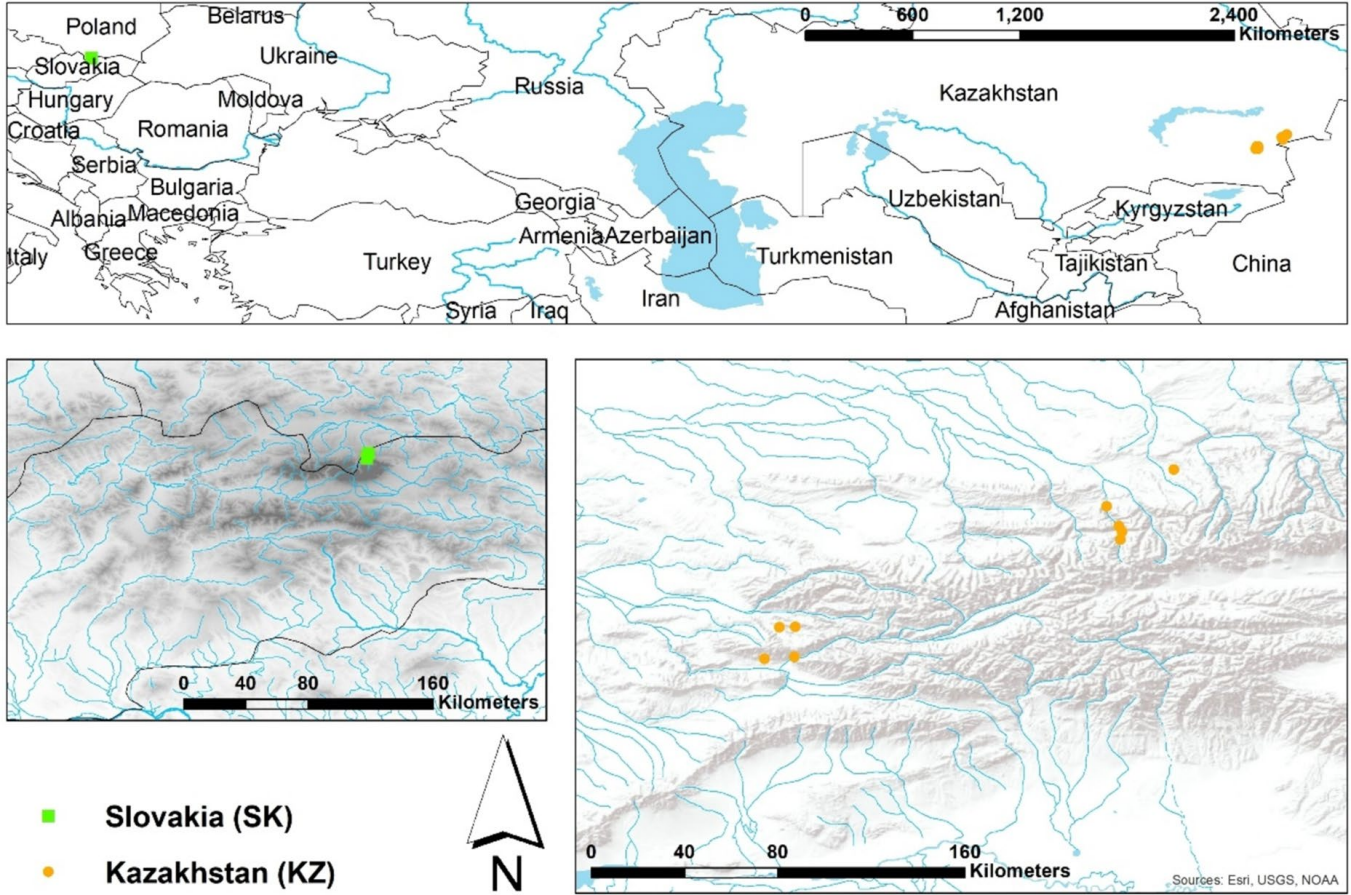
Study sites where Ephemeroptera and Plecoptera were collected

For this study, we focused on Ephemeroptera and Plecoptera species that inhabit mountain streams in the northern hemisphere. Most Ephemeroptera nymphs are herbivores with two main categories of feeding behavior: collectors and scrapers (Sartori and Brittain 2015). Within the Ephemeroptera, we focused primarily on the family Heptageniidae (flat-headed mayflies), which are generally considered to be scrapers (Merritt et al. 2017). Plecoptera nymphs are known to be predators, feeding on other aquatic insects. Species from these two orders were collected from mountain streams in Slovakia and Kazakhstan (Fig. 1).

In Slovakia, we have been continuously (monthly) monitoring trace elements concentrations in freshwater benthic macroinvertebrates in the mountain stream Javorinka (Tatra Mountains, Western Carpathians) since 2019. This mountain stream, fed by precipitation, is situated in a national park with strict protection and is minimally affected by tourism (limited to movement on tourist trails). The monitored species included *Ecdyonurus venosus* and *Perla grandis*. In Kazakhstan, in cooperation with Zhetysu University in Taldykorgan, we investigated trace elements concentrations in the bodies of freshwater benthic macroinvertebrates at nine sites in mountain streams/tributaries of the main rivers in the Zhetysu region. These rivers include the Koksu, Karatal, Lepsy, and Aksu, which originate in the Dzungarian Alatau. All sites were selected in parts of streams with no industrial activity in the upper catchment. In these sites, we collected species from the *Ecdyonurus* and *Perla genera*. All samples from Kazakhstan were from mountain streams primarily fed by mountain glaciers. These sites share common features such as poorly developed riparian vegetation, extensive seasonal grazing (horses, sheep, and cows), and riverbeds formed by deposits of gravel, stones, and boulders.

Benthic macroinvertebrates were randomly sampled across approximately 30 m of each investigated mountain stream/river. In total, nine sites across four streams/rivers were investigated in September 2022 in Kazakhstan. In Slovakia, samples were collected from two specific sites in the Javorinka stream, monthly from 2019 to 2021. From these monthly samples, we selected only those from autumn months. The minimum distance between the two sites was set to 1 km. Benthic macroinvertebrates were collected using the “kicking technique” method (Frost et al. 1971). A hydrobiology D-net with a 0.25 mm mesh size was used. Samples were stored in clean glass bottles with stream water from the site and fixed with a 4% formaldehyde solution (Leuven et al. 1985). Immediately after fieldwork, the nymphs were separated from the detritus, rinsed with deionized water, and identified by stereomicroscope to the order level: Ephemeroptera and Plecoptera. Samples were dried at 50 °C and then homogenized using a hand mortar. The homogenized samples were placed in a plastic vial of the same volume and analyzed by a handheld ED-XRF spectrometer with XRF WorkStation (ED-XRF DELTA, Olympus, Innov-x Systems, USA).

The ED-XRF spectrometer is commonly used for the quantitative identification of trace elements such as P, S, Cl, K, Ca, Ti, Cr, Mn, Fe, Co, Ni, Cu, Zn, As, Se, Rb, Sr, Zr, Mo, Ag, Cd, Sn, Sb, Ba, Hg, and Pb. The detection limits vary for different trace elements as described in the Delta XRF manual (Innov-X Systems 2018). Despite the detection limits, this method allows for accurate comparison of trace elements concentrations in samples. Each sample was measured nine times by exposure to the X-ray beam for 80 s, and the measured results were averaged (in ppm). Additionally, randomly selected samples were remeasured using the same procedure. The relative standard deviation between measured values of samples was below 5%. Trace elements detected with 20% (or more) of cases below the limit of detection (<LOD) were excluded from the assessment. Finally, only S, K, Ca, Cr, Mn, Fe, Cu, Zn, Rb, Mo, and Pb were processed for statistical evaluation.

At each sampling site, physical parameters such as pH, water temperature (°C), concentrations of total dissolved solids (TDS mg/l), and dissolved oxygen (mg/l) were measured in situ using a portable multimeter (MultiLine 3430, WTW GmbH, Weilheim, Germany) with compatible probes: IDS pH electrode SenTixR 940-3, TDS electrode TetraCon 925-3, and an optic oxygen electrode FDO 925-3. All in situ measurements were repeated three times and then averaged.

Statistical analysis was performed using the Statistica 12 software (TIBCO Software Inc., Palo Alto, CA, USA). Non-parametric tests were used to compare differences between data groups due to the small sample size for each group (*n* < 30) and the non-normal distribution of the data (Shapiro-Wilk test, *p* < 0.01). For each trace element, the differences in concentrations between groups of freshwater benthic macroinvertebrates (Ephemeroptera as scrapers vs. Plecoptera as predators) were tested using the Mann-Whitney *U* test (M-W U test). Sites (Slovakia – SK and Kazakhstan – KZ) were also compared using the M-W *U* test. The significance level was set at *p* < 0.05. Principal components analysis (PCA) was used to reduce individual variables (trace elements) to new components that could better explain the correlation and accumulation of trace elements in the two groups of freshwater benthic macroinvertebrates. Principal components (PC) with eigenvalues greater than one were explained. The PC coordinates of the cases (based on correlations) divided among groups of freshwater benthic macroinvertebrates and sites were further tested using the Kruskal-Wallis *H* test. Finally, relationships between levels of trace elements in the bodies of freshwater benthic macroinvertebrates and water parameters were analyzed using correlation analysis. This analysis included principal component scores and water parameters such as temperature, pH, total dissolved solids, and dissolved oxygen content. Data were analyzed first as a whole and then separately for the two groups of benthic macroinvertebrates.

## Results

A comparison of data for all trace elements between our two groups of benthic macroinvertebrates revealed only a small number of trace elements that significantly differed between the scraper and predator groups in the study areas (Table 1). Concentrations of Ti, Fe, and Sr were significantly higher in predators than in scrapers from Kazakhstan, and concentrations of Cu were significantly higher in predators than in scrapers from Slovakia. Despite no significant differences, trace elements such as S, K, Ca, Cr, Mn, Fe, Rb, Mo, Ba, and Pb were measured in higher concentrations in scrapers than in predators from Slovakia, while Ti, Zn, and Sr were measured in higher concentrations in predators than in scrapers. In Kazakhstan, only concentrations of S, Cr, Rb, and Pb were higher in scrapers than in predators, and concentrations of trace elements such as Cl, K, Ca, Mn, Cu, Zn, Mo, and Ba were higher in predators than in scrapers.

**Table 1:** Mean values and differences (by Mann-Whitney *U* test) of physical parameters of the water and trace elements measured in bodies of freshwater benthic macroinvertebrates (Scrapers — Ephemeroptera, Predator — Plecoptera) collected from mountain streams in Slovakia and Kazakhstan (significantly high mean values between sites are in bold and significantly high mean values between groups are in bold–italic; significant differences *p* < 0.05)

	Groups	All sites		Slovakia		Kazakhstan		M-W U	M-W U Predators vs. Scrapers in		
		*N*	Mean (SE)	*N*	Mean (SE)	*N*	Mean (SE)	Sites	All	SK	KZ
Trace elements in freshwater benthic macroinvertebrate											
S [ppm]	All	51	9243.42 (848.19)	34	9556.92 (1250.56)	17	8616.41 (506.66)	0.390			
	Predators	23	7869.68 (1012.54)	14	7719.55 (1639.64)	9	8103.22 (631.03)	0.089	0.068	0.104	0.290
	Scrapers	28	10371.85 (1279.02)	20	10843.08 (1765.96)	8	9193.75 (803.07)	0.879			
Cl [ppm]	All	51	10673.72 (614.86)	34	9247.55 (680.70)	17	**13526.06 (932.84)**	**0.001**			
	Predators	23	11369.27 (914.37)	14	9226.51 (1102.21)	9	**14702.44 (723.42)**	**0.001**	0.264	0.889	0.149
	Scrapers	28	10102.38 (829.92)	20	9262.28 (886.68)	8	12202.63 (1755.33)	0.154			
K [ppm]	All	51	25503.05 (1702.79)	34	20003.75 (1866.63)	17	**36501.65 (1212.09)**	**0.001**			
	Predators	23	26880.67 (2714.63)	14	19218.39 (2831.42)	9	**38799.78 (1533.03)**	**0.001**	0.405	0.861	0.054
	Scrapers	28	24371.42 (2177.74)	20	20553.49 (2531.70)	8	**33916.25 (1520.04)**	**0.004**			
Ca [ppm]	All	51	10754.47 (1045.28)	34	10859.06 (1438.43)	17	10545.29 (1305.41)	0.646			
	Predators	23	11146.99 (1258.96)	14	10223.05 (1625.54)	9	12584.22 (2008.81)	0.314	0.188	0.637	0.102
	Scrapers	28	10432.05 (1617.40)	20	11304.27 (2195.08)	8	8251.5 (1291.88)	0.760			
Ti [ppm]	All	35	483.97 (88.16)	18	166.88 (19.78)	17	**819.71 (140.95)**	**0.001**			
	Predators	13	***821.51 (190.35)***	4	207.17 (66.78)	9	***1094.56 (217.89)***	**0.021**	***0.007***	0.288	***0.027***
	Scrapers	22	284.51 (51.81)	14	155.37 (17.83)	8	**510.5 (98.23)**	**0.002**			
Cr [ppm]	All	51	70.98 (6.90)	34	68.8 (9.85)	17	**75.35 (6.62)**	**0.026**			
	Predators	23	58.63 (5.23)	14	49.25 (6.96)	9	**73.22 (5.12)**	**0.017**	0.67	0.234	0.564
	Scrapers	28	81.13 (11.56)	20	82.48 (15.47)	8	77.75 (13.33)	0.387			
Mn [ppm]	All	51	238.25 (20.49)	34	194.12 (13.56)	17	326.53 (49.48)	**0.001**			
	Predators	23	263.83 (42.94)	14	183.79 (27.78)	9	**388.33 (88.46)**	**0.006**	0.609	0.327	0.092
	Scrapers	28	217.25 (12.09)	20	201.35 (12.91)	8	**257 (22.97)**	**0.039**			
Fe [ppm]	All	51	5018.79 (850.92)	34	1810.89 (235.38)	17	**11434.59 (1643.34)**	**0.001**			
	Predators	23	6686.01 (1659.67)	14	1595.67 (399.38)	9	***14604.33 (2465.56)***	**0.001**	0.663	0.248	***0.043***
	Scrapers	28	3649.29 (666.81)	20	1961.55 (290.34)	8	**7868.63 (1367.45)**	**0.001**			
Cu [ppm]	All	50	74.2 (4.11)	33	70.39 (5.34)	17	81.59 (6.03)	0.190			
	Predators	23	***87.09 (6.37)***	14	***85.23 (9.42)***	9	90 (7.68)	0.950	***0.002***	***0.017***	0.163
	Scrapers	27	63.21 (4.43)	19	59.46 (5.01)	8	72.13 (8.77)	0.184			
Zn [ppm]	All	51	444.99 (65.03)	34	379.28 (45.18)	17	576.41 (172.08)	0.646			
	Predators	23	***515.37 (113.97)***	14	418.25 (64.76)	9	666.44 (275.66)	0.900	***0.041***	0.208	0.068
	Scrapers	28	387.18 (72.78)	20	352 (62.63)	8	475.13 (208.21)	0.919			
Rb [ppm]	All	48	24.33 (1.96)	31	16.98 (1.55)	17	**37.74 (2.50)**	**0.001**			
	Predators	22	23.37 (2.42)	13	16.13 (1.89)	9	**33.84 (2.59)**	**0.001**	0.959	0.952	0.149
	Scrapers	26	25.14 (3.02)	18	17.59 (2.33)	8	**42.11 (4.07)**	**0.001**			
Sr [ppm]	All	44	12.43 (1.50)	28	11.89 (2.13)	16	13.38 (1.86)	0.188			
	Predators	21	***14.45 (1.59)***	12	12.52 (2.08)	9	***17.03 (2.31)***	0.082	***0.006***	0.086	***0.017***
	Scrapers	23	10.59 (2.46)	16	11.43 (3.44)	7	8.67 (1.99)	0.526			
Mo [ppm]	All	43	6.61 (0.37)	26	5.94 (0.54)	17	**7.65 (0.32)**	**0.003**			
	Predators	20	6.44 (0.43)	11	5.3 (0.49)	9	**7.83 (0.43)**	**0.003**	0.679	0.287	0.700
	Scrapers	23	6.76 (0.58)	15	6.4 (0.85)	8	7.44 (0.51)	0.197			
Ba [ppm]	All	48	182.36 (13.03)	31	139.75 (8.96)	17	**260.06 (23.46)**	**0.001**			
	Predators	22	200.13 (24.87)	13	130 (17.29)	9	**301.44 (33.92)**	**0.001**	0.548	0.262	0.124
	Scrapers	26	167.32 (11.41)	18	146.8 (9.20)	8	**213.5 (24.66)**	**0.018**			
Pb [ppm]	All	45	14.82 (1.02)	28	12.67 (0.59)	17	**18.35 (2.31)**	**0.006**			
	Predators	23	14.67 (1.52)	14	12.61 (0.38)	9	**17.89 (3.72)**	**0.047**	0.982	0.662	0.47
	Scrapers	22	14.97 (1.37)	14	12.74 (1.13)	8	18.88 (2.82)	0.052			
Physicochemical parameters of stream water											
T [°C]	All	51	6.96 (0.39)	34	5.89 (0.34)	17	**9.11 (0.73)**	**0.001**			
	Predators	23	7.1 (0.67)	14	5.59 (0.54)	9	**9.44 (1.12)**	**0.006**	0.992	0.473	0.700
	Scrapers	28	6.85 (0.47)	20	6.1 (0.44)	8	**8.73 (0.97)**	**0.031**			
pH	All	51	8.27 (0.06)	34	8.15 (0.08)	17	8.51 (0.08)	0.079			
	Predators	23	8.31 (0.09)	14	8.19 (0.12)	9	8.5 (0.11)	0.208	0.762	0.753	0.923
	Scrapers	28	8.24 (0.09)	20	8.13 (0.11)	8	8.52 (0.13)	0.222			
TDS [mg/l]	All	51	190.19 (7.06)	34	**196.84 (4.61)**	17	176.88 (19.08)	**0.005**			
	Predators	23	193.49 (13.04)	14	195.88 (7.76)	9	189.78 (32.26)	0.123	0.902	0.889	0.700
	Scrapers	28	187.48 (7.34)	20	**197.52 (5.81)**	8	162.38 (19.30)	**0.015**			
O_2_ [mg/l]	All	51	10.92 (0.16)	34	**11.6 (0.11)**	17	9.58 (0.13)	**0.001**			
	Predators	23	10.84 (0.26)	14	**11.68 (0.17)**	9	9.52 (0.19)	**0.001**	0.656	0.612	0.700
	Scrapers	28	11 (0.20)	20	**11.54 (0.14)**	8	9.63 (0.18)	**0.001**			

The distribution of trace elements in this way indicates differences between samples from Slovakia and Kazakhstan. In general, the concentrations of all trace elements were higher in predators from Kazakhstan than in Slovakia, with particularly significant differences observed for Cl, K, Ti, Cr, Mn, Fe, Rb, Mo, Ba, and Pb. Comparing scrapers from Slovakia and Kazakhstan showed insignificantly higher concentrations of S, Ca, Cr, and Sr in scrapers from Slovakia, while concentrations of other trace elements, especially K, Ti, Mn, Fe, Rb, and Ba, were significantly higher in scrapers from Kazakhstan. In summary, concentrations of trace elements such as S, Ca, Cu, Sr, and Zn were indifferent in both areas, as well as Cl, Cr, Mo, and Pb in scrapers. When comparing the parameters of the streams where the samples were collected, we found that the samples in Slovakia were collected in colder conditions with significantly higher concentrations of oxygen and total dissolved solids (TDS) than in Kazakhstan.

**Fig. 2:**
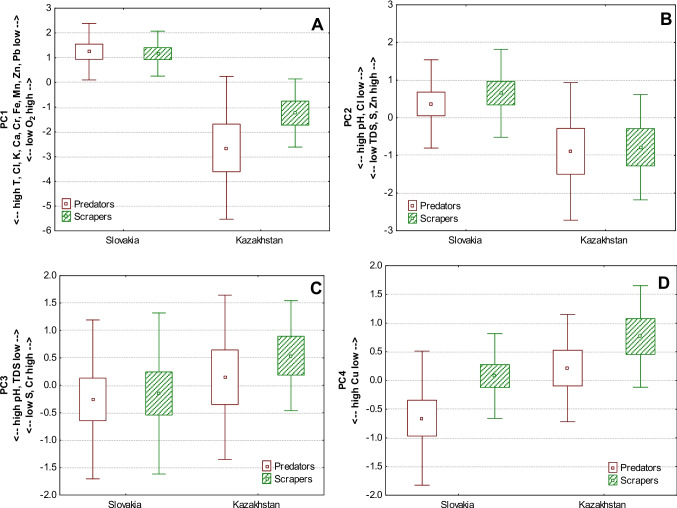
Principal component coordinates for the trace elements measured in scrapers and predators collected from mountain streams in Slovakia and Kazakhstan. **A** PC1 represented by general loading of trace elements [Scrapers vs sites: KW-H (1, 22) = 11.646, *p* = 0.0006; Predators vs sites: KW-H (1, 23) = 14.2857, *p* = 0.0002; Scrapers vs Predators from Slovakia: KW-H (1, 28) = 0.0021, *p* = 0.9634; Scrapers vs Predators from Kazakhstan: KW-H (1, 17) = 0.9259, *p* = 0.3359].** B** PC2 explained high concentrations of S and Zn in benthic macroinvertebrates which were collected from streams with high TDS and low pH [Scrapers vs sites: KW-H (1, 22) = 7.8307, *p* = 0.0051; Predators vs sites: KW-H (1, 23) = 7.3373, *p* = 0.0068; Scrapers vs Predators from Slovakia: KW-H (1, 28) = 1.0218, *p* = 0.3121; Scrapers vs Predators from Kazakhstan: KW-H (1, 17) = 1.5648, p = 0.2110].** C** PC3 explained high concentrations of S and Cr in benthic macroinvertebrates which were collected from streams with low pH and TDS [Scrapers vs sites: KW-H (1, 22) = 2.6832, *p* = 0.1014; Predators vs sites: KW-H (1, 23) = 1.1468, *p* = 0.2842; Scrapers vs Predators from Slovakia: KW-H (1, 28) = 0.1351, *p* = 0.7132; Scrapers vs Predators from Kazakhstan: KW-H (1, 17) = 0.1481, *p* = 0.7003].** D** PC4 described high concentrations of Cu in benthic macroinvertebrates [Scrapers vs sites: KW-H (1, 22) = 2.2547, *p* = 0.1332; Predators vs sites: KW-H (1, 23) = 3.5714, *p* = 0.0588; Scrapers vs Predators from Slovakia: KW-H (1, 28) = 4.4673, *p* = 0.0346; Scrapers vs Predators from Kazakhstan: KW-H (1, 17) = 1.3333, *p* = 0.2482]; (middle points — means; boxes — +/− standard errors of mean; whiskers — +/− standard deviations of mean)

These less significant differences between groups and more significant differences between sites prompted us to use principal component analysis (PCA), which allowed us to reduce individual variables to new components that could better explain the correlation and concentrations of trace elements in these two macroinvertebrate groups. For PCA, we selected only trace elements that were successfully measured in almost all samples. The resulting principal components (PC) with eigenvalues up to one were further processed (Table 2). Their PC coordinates (based on correlations) were compared in our two macroinvertebrate groups and sites. The first component, PC1 (36.24% of the total variance), is primarily associated with high temperature, Cl, K, Ca, Cr, Mn, Fe, Zn, Pb, and low O_2_, showing a general higher loading of trace elements (consistent for both benthic macroinvertebrate groups: scrapers and predators) in warmer conditions with less oxygen in the stream. Despite insignificant differences between predators and scrapers, this factor indicated that a higher general loading of trace elements is stronger in Kazakhstan than in Slovakia. However, predators in Kazakhstan had higher variability of the factor than scrapers (Fig. 2A). The second component, PC2 (15.59% of the total variance), is strongly associated with high TDS, S, Zn, and low pH, Cl, suggesting that samples (both scrapers and predators) with high concentrations of S and Zn were collected in sites with high concentrations of total dissolved solids and low pH (Fig. 2B). The significant variations observed in PC1 and PC2 across sites highlight the influence of local environmental conditions, including water chemistry, on the bioaccumulation patterns of trace elements in these organisms.

**Table 2:** Principal components of trace elements measured in bodies of freshwater benthic macroinvertebrates collected from Slovakia and Kazakhstan (factor coordinates greater than 0.4 or less than −0.4 in each PC column are in bold; valid number of samples: 45)

	PC1	PC2	PC3	PC4
T [°C]	**−0.774**	**−**0.021	**−**0.135	0.141
pH	**−**0.185	**−0.536**	**−0.587**	0.231
TDS [mg/l]	−0.340	**0.675**	**−0.458**	−0.132
O_2_ [mg/l]	**0.796**	0.361	−0.048	−0.205
S [ppm]	−0.103	**0.556**	**0.720**	0.110
Cl [ppm]	**−0.571**	**−0.565**	0.179	−0.321
K [ppm]	**−0.691**	−0.371	0.388	−0.008
Ca [ppm]	**−0.434**	0.214	−0.076	−0.283
Cr [ppm]	**−0.499**	0.185	**0.658**	0.055
Mn [ppm]	**−0.837**	0.274	−0.190	0.101
Fe [ppm]	**−0.780**	−0.228	0.039	0.303
Cu [ppm]	−0.351	−0.212	0.048	**−0.801**
Zn [ppm]	**−0.688**	**0.429**	−0.392	−0.145
Pb [ppm]	**−0.731**	0.325	−0.038	0.086
Eigenvalue	5.07	2.18	1.91	1.10
Total variance %	36.24	15.59	13.64	7.88

The third component, PC3 (13.64% of the total variance), is associated with high S, Cr and low pH, TDS, suggesting that concentrations of S and Cr may be high in both benthic macroinvertebrate groups when pH and concentrations of TDS are low in the stream. This interplay between S and Cr in acidic environments and their combined impact on benthic macroinvertebrates may not be fully influenced by site-specific factors (Fig. 2C). The fourth component, PC4 (7.88% of the total variance), is mainly associated with a high concentration of Cu in benthic macroinvertebrates. Significant differences showed that predators, particularly from Slovakia, had higher concentrations of Cu than scrapers (Fig. 2D). The PCA results indicate distinct patterns of trace elements accumulation and water parameter influences in freshwater benthic macroinvertebrates between the study sites in Slovakia and Kazakhstan. These findings suggest that both biotic (feeding strategies) and abiotic (geographical and environmental conditions) factors might play a critical role in the trace elements dynamics of freshwater ecosystems.

## Discussion

Mountain streams and rivers provide habitats for well-adapted species of freshwater benthic macroinvertebrates. Based on their different feeding preferences, we expected different trace elements accumulation in different macroinvertebrate groups (Pastorino et al. 2019; 2020b). Our investigated trophic guilds, scrapers and predators, from both study areas showed insignificant differences in accumulated levels of trace elements (S, Cl, K, Ca, Ti, Cr, Mn, Fe, Cu, Zn, Rb, Sr, Mo, Ba, and Pb), except for Fe, Ti, and Sr in Kazakhstan and Cu in Slovakia. On the other hand, the differences in trace elements concentrations between the study areas were highly variable. This can be generally explained by different environmental conditions including the physicochemical properties of water and sediments (Brezonik et al. 1991; Miranda et al. 2021).

In summary, regardless of significance, Slovak scrapers had higher levels of S, K, Ca, Cr, Mn, Fe, Rb, Mo, Ba, and Pb, whereas Kazakhstan scrapers had only higher levels of S, Cr, Rb, and Pb than predators. On the other hand, predators had higher levels of Ti, Cu, Zn, and Sr in Slovakia, but predators from Kazakhstan had higher levels of Cl, K, Ca, Ti, Mn, Fe, Cu, Zn, Sr, Mo, and Ba than scrapers. Despite the insignificant differences, we can consider that trace elements such as S, Cr, Rb, and Pb were accumulated at higher levels in scrapers, and trace elements such as Ti, Cu, Zn, and Sr in predators in both study areas. Santoro et al. (2009) noticed that varied behavior of different freshwater benthic macroinvertebrate groups (according to their functional feeding preferences: collector-gatherers, predators, and filter-feeders) can explain the bioaccumulation of different trace elements (e.g., heavy metals such as As, Cr, Zn). However, they also mentioned that all kinds of organisms (regardless of their behavior or feeding preferences), take up increasing amounts of trace elements as sediment contamination increases. Because the investigated streams in Kazakhstan are mostly fed by glaciers, this environment might be richer in available trace elements for accumulation. In this sense, the generally higher levels of accumulated trace elements in freshwater benthic macroinvertebrates from Kazakhstan could be attributed to a combination of environmental, geological, ecological, and physiological factors specific to that region and the investigated streams. Despite this, some trace elements were higher in Slovakia than in Kazakhstan, specifically S, Ca, Cr, and Sr, however only in scrapers. This means that these trace elements may be more available in basal resources for scrapers in Slovakia.

Variability in trace elements bioaccumulation is widely confirmed (Goodyear and McNeill 1999; Luoma and Rainbow 2005). In a study by Pastorino et al. (2020b), scrapers accumulated higher amounts of trace elements, including Cr, Fe, Mn, Pb, and Sr compared to other functional feeding groups (e.g., predators or filterers). Some trace elements, including Ba, Ti, and Zn, were high in predators. Compared to our results, without considering significance, we can confirm, in alignment with Pastorino et al. (2020b), a higher concentration of Cr and Pb for scrapers (additionally also Fe and Mn in Slovak scrapers), and Ti and Zn for predators. However, mean values of Fe (significantly) and Mn from Kazakhstan samples were higher for predators, probably due to the high amount of these trace elements in the investigated streams or in their basal resources or prey. This higher amount may also be caused by pollution, as in very polluted areas there may be a greater difference in trace elements concentrations between feeding groups, particularly in the case of metals (Burrows and Whitton 1983). In our case, significant differences were also observed specifically for Ti, Sr, and Cu. Arnold et al. (2021) investigated several macroinvertebrate genera (Gammarus, Baetis, Leuctra, Hydropsyche, and Rhyacophila) in Great Britain and identified differences between predatory and non-predatory aquatic insects. Additionally, they pointed out that trace elements such as Fe, Mn, Cu, and Zn were relatively bioavailable across all sites, and some of them, specifically Mn, Cu, and Pb, had consistent correlations between most insects’ guilds, despite different feeding styles or dietary preferences. Consistent correlations of Cu and Pb between individual feeding guilds were also confirmed in the review by Goodyear and McNeill (1999), which investigated the biomagnification of Zn, Cu, and Pb, and their intercept differences from sediment and water. It is not surprising because Cu and Pb are often bound to Fe and Mn oxyhydroxides or bound to organic matter.

Physicochemical conditions of streams may influence trace elements bioavailability (Wojtkowska et al. 2016; Keshavarzifard et al. 2019; Iordache et al. 2022; Li et al. 2023). Our results indicate that both scrapers (Ephemeroptera) and predators (Plecoptera) had higher levels of trace elements such as Cl, K, Ca, Cr, Fe, Mn, Zn, and Pb in their bodies at warmer sites with less oxygen (usually lower parts of the mountain streams), particularly in Kazakhstan. It is reasonable to assume that this is due to the higher volume of organic and inorganic matter in the lower parts of wider and slower-moving streams (otherwise, oxides of S, Fe, and Mn are good absorbents of metals like Pb). These trace elements might be more abundant also because they are easily mobilized from sediments (Miller et al. 1992; Horváth et al. 2013; Roig et al. 2016).

On the other hand, the results of our study have some limitations because all samples were collected in autumn, and it is generally known that there are certain seasonal variations in trace elements in the aquatic environment (Köck et al. 1996; Pastorino et al. 2020a), also in conjunction with feeding habits variations. For example, predators have slower gut clearance time and they consume less prey during colder conditions (Allan 1982). In addition, some proteins responsible for metal homeostasis and detoxification may also vary by season (Geffard et al. 2007). Additionally, our study did not include the collection of sediment samples, which could provide crucial information on the source and extent of trace elements contamination in the aquatic environment (Santoro et al. 2009). Sediment samples are essential for understanding the baseline levels of trace elements and their potential bioavailability to benthic macroinvertebrates. Without this data, our understanding of the full scope of trace elements bioaccumulation and its influencing factors remains incomplete. Further studies incorporating sediment analysis would be beneficial to address these gaps and provide a more comprehensive assessment of trace elements dynamics. The bioaccumulation of trace elements in sediments, water, or the food web is an extraordinarily complex issue. While this study identified several differences in trace elements accumulation between scrapers and predators, further research is warranted to elucidate the underlying mechanisms driving these differences. Investigating the dietary preferences, foraging behaviors, and habitat use of these macroinvertebrate groups could provide valuable insights into their respective roles in trace elements cycling within aquatic ecosystems and support evidence-based management and conservation efforts aimed at protecting these vital aquatic resources.

## Data Availability

Data sets generated during the current study are available from the corresponding author upon reasonable request.
